# Spatiotemporal simulation of sustainable development based on ecosystem services under climate change

**DOI:** 10.1371/journal.pone.0316605

**Published:** 2025-02-04

**Authors:** Bao Zhou, Guoping Chen, Junsan Zhao, Ying Yin

**Affiliations:** 1 College of Electronic and Information Engineering, West Anhui University, Luan, China; 2 Faculty of Land Resource Engineering, Kunming University of Science and Technology, Kunming, China; Zhejiang A&F University, CHINA

## Abstract

This study explores the spatiotemporal distribution characteristics of ecosystem services (ESs) in the karst region of southeastern Yunnan under the backdrop of climate change. The study innovatively calculates the sustainable development goals (SDG) index based on ecosystem services (ESs). It employs the patch-generating land use simulation (PLUS) model to simulate future land use changes (LUCs) and uses the integrated valuation of ecosystem services and tradeoffs (InVEST) model to assess ESs under different scenarios. This research systematically evaluates the ESs and SDGs in karst regions within the context of climate change. The results indicate that: (1) Under all three scenarios in 2035, the trend of LUCs in the karst area of southeastern Yunnan is highly consistent, though the intensity and spatial configuration vary significantly. The least reduction in arable land area occurs under the shared socioeconomic pathways (SSP) 126 scenario, while water bodies and construction land show varying degrees of increase; (2) Regarding ESs, both water yield and soil retention exhibit an increasing trend across all scenarios by 2035, with the most notable rise under SSP126. Conversely, habitat quality and carbon storage show a decline, with the smallest decrease also under SSP126; (3) Analyzing the SDG index, the overall value is low in 2020. In future scenarios, the SDG index increases in the southern part while decreasing in the eastern part, indicating significant differences in regional sustainable development potential. Hotspots under SSP126 and SSP245 are concentrated in the densely vegetated southwest and eastern edge areas, while cold spots are mainly found in the heavily human-impacted central Yunnan urban agglomeration and Wenshan City. This study systematically explores for the first time the spatiotemporal dynamics of ESs in the karst region of southeastern Yunnan under different climate scenarios. It provides scientific evidence for regional ecological protection and land use planning.

## 1. Introduction

Climate change represents one of the primary environmental challenges faced by global ecosystems [1]. In recent years, the ecological shifts triggered by global climate change have become a focal point of research. Climate change profoundly influences the structure and function of ecosystems by altering temperature, precipitation patterns, and the frequency of extreme weather events [2]. Such changes can lead to a loss of biodiversity, a decline in ecosystem services (ESs), and negative impacts on human societies [3]. For instance, the rise in global temperatures and the increased occurrence of extreme weather events due to climate change have destroyed habitats for many species, affecting the stability and function of ecosystems [4]. Additionally, climate change has disrupted global precipitation patterns, leading to droughts or flooding in various regions and exacerbating the vulnerability of ecosystems [5]. The karst region of southeastern Yunnan, one of China’s three major karst centers, boasts unique geographical and ecological characteristics, including complex terrain, diverse biotic communities, and a distinctive hydrological system [6]. The region primarily experiences tropical and subtropical monsoon climates, with annual precipitation ranging from 1000 to 2000 millimeters and an average annual temperature between 14 and 24 degrees Celsius [7]. However, this region also faces challenges from climate change, significantly impacting ESs.

Presently, research on climate change primarily focuses on concepts [8], formation mechanisms [9], spatiotemporal evolution [10], trends [11], and comprehensive governance [12]. Some scholars have also conducted studies on extracting climate change information, exploring the interrelations between climate change and natural and anthropogenic factors [13], simulating future climate change scenarios [14], and introducing key technologies and experiences for climate change mitigation [15]. Chang et al. (2024) examined the interactions among landscape patterns, climate change, and ESs, highlighting their impacts on regional sustainability [16]. Assessing the effects of climate change and land use changes (LUCs) on ESs through these models can effectively evaluate the impacts on the supply and function of ESs [17]. Climate change, by altering temperature, precipitation, and extreme weather events, influences the spatial distribution and function of ESs [18]. For example, Wen et al. (2024) investigated the spatial and seasonal impacts of future climate and LUCs on ESs, finding that these changes significantly affect the supply and function of regional ESs [19]. The temperature rise and precipitation changes caused by climate change lead to a loss of biodiversity, ecosystem degradation, and a weakening of ES functions [20]. These changes further impact the regulatory and productive capacities of ecosystems, posing a threat to human well-being [21]. In studies examining the relationship between ESs and sustainability, there is a common focus on the impacts of climate change on ESs, such as biodiversity, regulatory functions of ecosystems, and threats to human well-being [22]. However, despite providing valuable insights, there are still some shortcomings. For instance, many studies concentrate on single ecosystems or specific regions, lacking a comprehensive consideration of interactions between different ecosystems [23]. Additionally, existing research often fails to adequately explore how changes in ESs under different socio-economic contexts affect the achievement of SDGs [24]. This study aims to address these gaps by comprehensively considering the dynamic relationships among climate change, LUCs, and ESs. Unlike existing research, this study not only focuses on changes in the supply and function of ESs but also delves into how these changes impact sustainability across different regions and socio-economic contexts. Employing an interdisciplinary approach, combining socio-economic data with ecological models, this study strives to comprehensively assess the critical role of ESs in achieving sustainable development goals (SDGs).

Existing research primarily focuses on the assessment of ESs in karst regions [25], but studies comprehensively addressing the impacts of climate change are relatively limited. This study innovatively integrates multi-source data and models to systematically evaluate the spatial distribution and dynamic changes of ESs and SDGs under the backdrop of climate change in the karst region of southeastern Yunnan. This fills the current research gap and provides scientific evidence for the management of ESs and sustainable development strategies in the region. This has practical significance for the management of ESs and the optimal allocation of land resources. By revealing the mechanisms through which climate change impacts ESs, the study offers scientific evidence for ecological protection and sustainable development strategies in the region. Additionally, this research can provide a referential framework and methods for regions with similar geographical environments.

## 2. Materials and methods

### 2.1. Study area

**Fig 1:**
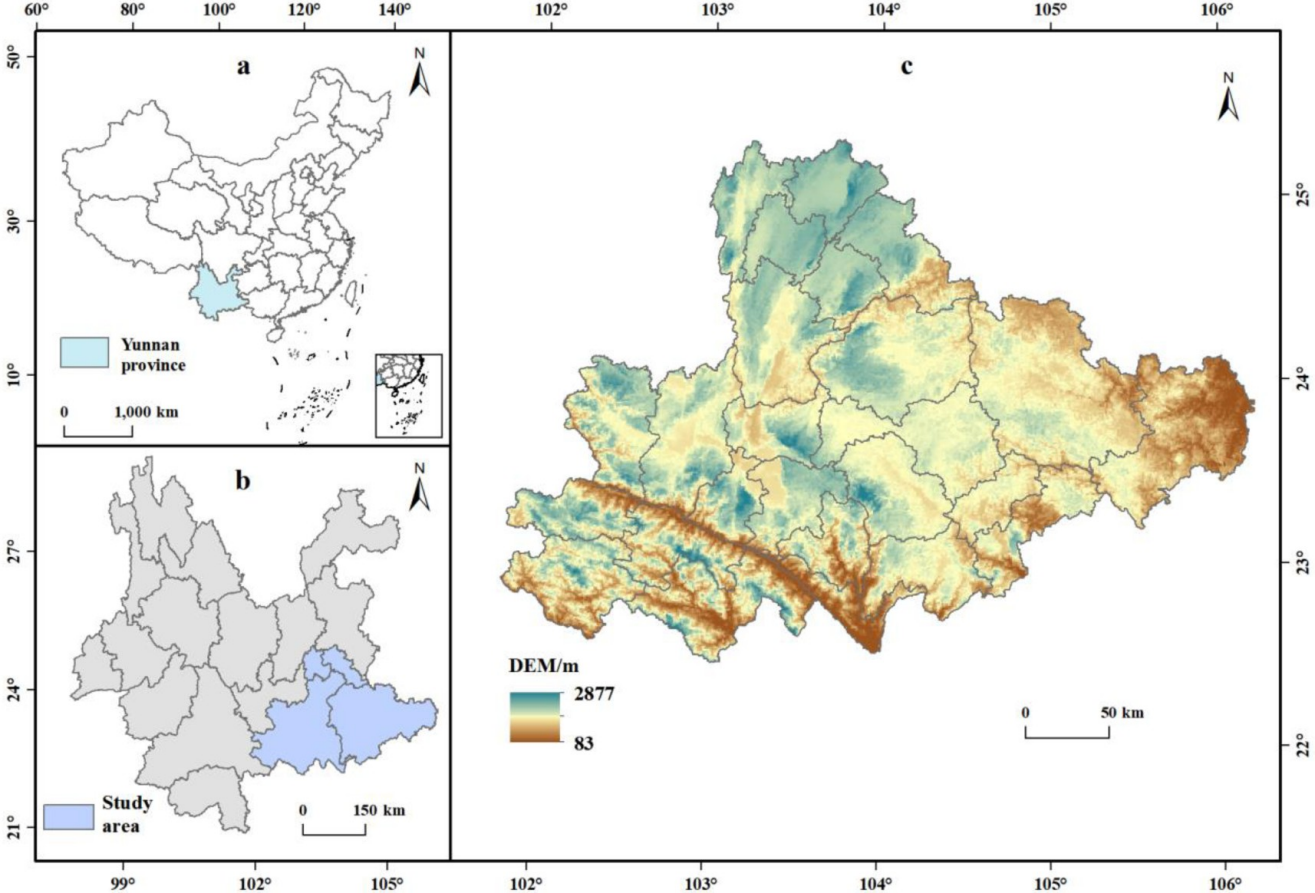
Study area.

The southeastern Yunnan karst region is located in the southeastern part of Yunnan Province (Fig 1), encompassing 25 counties across four prefectural-level administrative areas: Wenshan Prefecture (entire area), Honghe Prefecture (entire area), Qujing City (Shizong and Luoping counties), and Kunming City (Shilin and Yiliang counties). The region features a complex terrain with elevations ranging from 73 to 3056 meters, primarily consisting of mountainous hills and karst landforms with significant topographical relief and steep slopes. The climate is characterized by subtropical and temperate plateau monsoon conditions, with ample sunlight, uneven rainfall distribution, and high evaporation rates. Annual average precipitation ranges from 811 to 2000 millimeters, and the long-term average temperature varies between 9.5 to 24.0°C. The study area is located in the southeastern Yunnan rocky desertification zone, with widespread rocky desertification and potential rocky desertification land. The region faces severe issues of soil erosion, land degradation, and water scarcity, which significantly impact biodiversity and ecosystem stability. The southern part serves as a border ecological barrier zone, with extensive tropical rainforest coverage, high vegetation density, rich biodiversity, and relatively high ecological quality.

### 2.2. Data sources and processing

1. Land use data and normalized difference vegetation index (NDVI): These data were obtained from the Resource and Environment Data Center of the Chinese Academy of Sciences (https://www.resdc.cn/), with a spatial resolution of 30m for the years 2000, 2005, 2010, 2015, and 2020. (2) The 30m resolution digital elevation model (DEM) data was sourced from Geospatial Data Cloud (http://www.gscloud.cn). Slope data were calculated from the DEM using ArcGIS 10.8, also at a spatial resolution of 30m. Data on soil erodibility, root depth, and plant water capacity were obtained from the International Soil Reference and Information Centre (https://data.isric.org), with a resolution of 1km. The temporal resolution of these data is static, as they do not change frequently over short periods. (3) Social data: Population and gross domestic product (GDP) data were sourced from the Resource and Environment Science Data Center (https://www.resdc.cn/), with the year being 2020. The distance to railways, water sources, and roads was determined using spatial analysis tools in ArcGIS 10.8. (4) Meteorological data: These were obtained from the National Earth System Science Data Center (http://www.geodata.cn) and include precipitation, temperature, and evapotranspiration, with a monthly temporal resolution. The rainfall erosion factor was calculated using daily rainfall data. Future climate scenarios were downloaded from the Intergovernmental Panel on Climate Change (IPCC) (https://esgf-node.llnl.gov).

To ensure data quality, measures such as outlier correction and consistency checks were implemented. Specifically, the meteorological data underwent downscaling correction. For all raster data, resampling was performed using ArcGIS 10.8 to standardize the resolution to 30 meters. To meet the input requirements of the models, all data were projected onto the Krasovsky_1940_Albers coordinate system. The patch-generating land use simulation (PLUS) model was used to simulate future LUCs, while the integrated valuation of ecosystem services and tradeoffs (InVEST) model was utilized to assess ESs under different scenarios. The selection of these methods provided robust technical support for the research.

### 2.3. Land use and climate change scenario modeling settings

Based on the research objectives, future climate data under the shared socioeconomic pathways (SSPs) combined with representative concentration pathways (RCPs) scenarios were integrated with future land use scenarios [26]. Three scenarios were established: SSP126 Sustainable Development, SSP245 Natural Growth, and SSP585 Urban Expansion, to simulate LUC distribution characteristics by 2035 under different scenarios [27]. These three scenarios encompass a broad range of climate change pathways, from low emissions to high emissions, and are representative of various conditions. Additionally, these scenarios reflect different socioeconomic development patterns, thereby providing a comprehensive analytical perspective for the research.

1. SSP126 sustainable development scenario [28]. Under the SSP126 (low forcing, low emissions for sustainable development pathways) climate change model, the focus is on high ecological benefits, restricting the conversion of high-grade ecological land to lower grades, and controlling the transformation of ecological land into production and living areas. The probability of conversion from other lands to forests, grasslands, and water bodies is increased, promoting an overall improvement in the ecological landscape. This scenario represents an ideal sustainable development pathway that emphasizes ecological protection and the use of renewable energy.
2. SSP245 natural growth scenario. In the SSP245 (moderate forcing, moderate emissions for intermediate development pathways) climate change model, LUCs from 2000–2020 were followed without setting conversion probabilities between different land types [29]. This scenario maintains the existing development trend without considering future planning or special restrictions, allowing all land types except construction and water bodies to convert into each other. This scenario represents a balanced state that neither excessively emphasizes ecological protection nor completely disregards economic development, reflecting the actual development patterns and realities of many countries today.
3. SSP585 urban expansion scenario [30]. Under the SSP585 (high forcing, high emissions for unbalanced development pathways) climate change model, the emphasis is on economic development, with high-economic-benefit land designated as high-grade. Conversion follows a principle from low to high, prioritizing the needs of urban residents for production, business, residence, and recreational land when conflicts arise between ecological and production/living land uses, aiming for maximum economic benefits. This scenario represents a pathway that is highly dependent on fossil fuels and rapid urbanization, reflecting the pressures that may be placed on the environment and ecosystems in the context of economic prioritization.

### 2.4. PLUS model

The PLUS model [31], an improved cellular automata model built upon the FLUS model [32]. This approach allows for the prediction of LUCs in the study area by the year 2035 [33]. Using the Markov chain based on land use data from 2010 and 2015, combined with driving factor data, the areas of various land uses in the study area for 2020 were predicted [34]. The weighting settings for land use projections in 2035 across the three scenarios are shown in Table 1. The Kappa value of the PLUS model is 0.71, and the overall accuracy is 0.69, indicating good simulation performance. By integrating land use and driving factor data, the model provides a basis for predicting future LUC and for assessing ES and SDGs under climate change scenarios.

**Table 1:** Weighting settings for land use projections across multiple scenarios.

Land use type	SSP126	SSP245	SSP585
**Farmland weight**	0.2	0.25	0.15
**Forest weight**	0.4	0.3	0.2
**Grassland weight**	0.2	0.2	0.15
**Water weight**	0.05	0.05	0.05
**Built-up land weight**	0.1	0.15	0.4
**Unused land weight**	0.05	0.05	0.05

### 2.5. ESs assessment

This study utilized the InVEST model to calculate four types of ESs: water yield, carbon storage, habitat quality, and soil retention. The detailed parameters involved in the calculations can be found in the supplementary materials, and the simplified calculation formulas are as follows.

The specific formula for water yield is as follows [35, 36]:

$$Y_{ij}=(1-\frac{{AET}_{ij}}{P_{i}})\times P_{i}$$ (1)

$$\frac{{AET}_{ij}}{P_{i}}=\frac{1+\omega_{i}R_{i}}{1+\omega_{i}R_{i}+1/R_{i}}$$ (2)

$$\omega_{i}=Z\times \frac{{AWC}_{i}}{P_{i}}$$ (3)

$$R_{i}=\frac{k_{ij}\times AET}{P_{i}}$$ (4)

$${AWC}_{i}=M_{in}({MSD}_{i},{RD}_{i})\times {PAWC}_{i}$$ (5)

In the formula: Y_ij_ represents the annual water yield; AET_ij_ denotes the average annual evapotranspiration; P_i_ indicates the annual precipitation; ω_i_ is a non-physical, dimensionless parameter; R_i_ stands for the dryness index, which is also dimensionless; PAWC_i_ refers to the plant-available water content; k_ij_ represents the vegetation evapotranspiration coefficient; Z is the coefficient for seasonal factors; MSD_i_ denotes the maximum soil depth; and RD indicates the root depth.

The carbon storage formula is as follows [37–39]:

$$C_{tot}=C_{above}+C_{below}+C_{soil}+C_{dead}$$ (6)

In the formula: C_tot_ represents the total carbon storage; C_above_ denotes the aboveground carbon storage; C_below_ indicates the belowground carbon storage; C_soil_ refers to the soil carbon storage; and C_dead_ stands for the dead organic carbon storage.

Habitat quality is calculated as follows [40, 41]:

$$Q_{ij}=H_{j}\times (1-\frac{D_{ij}^{z}}{D_{ij}^{z}+k^{2}})$$ (7)

In the formula: Q_ij_ represents habitat quality; H_j_ denotes habitat suitability; D_ij_ indicates the level of land use pressure; k is the half-saturation constant; and Z is the normalization constant, set to 2.5.

The soil retention measurement formula is as follows [42, 43]:

SC=R×K×LS×C×P (8)

In the formula: SC denotes annual soil retention; R represents the rainfall erosivity factor; K indicates the soil erodibility factor; LS stands for the slope length-gradient factor; C denotes the cover management factor; P represents the conservation practice factor.

Calculation of total ESs. The entropy weight [44] method was used to determine the weight of each ES category (Table 2). These weights were then multiplied by the respective ES values and summed to obtain the total ES value [45, 46].

**Table 2:** Weights of each ecosystem service category during the study period.

Types of ES	2000	2005	2010	2015	2020	2035 SSP126	2035 SSP245	2035 SSP585
**Water yield**	0.31	0.32	0.33	0.34	0.35	0.36	0.35	0.34
**Soil retention**	0.35	0.34	0.33	0.32	0.31	0.3	0.31	0.32
**Habitat quality**	0.17	0.18	0.19	0.2	0.21	0.22	0.21	0.2
**Carbon storage**	0.17	0.16	0.15	0.14	0.13	0.12	0.13	0.14

### 2.6. Analysis of spatial agglomeration

We utilize Moran’s I index [47, 48] to evaluate spatial autocorrelation and create hot spot maps to visually represent the spatial distribution of ESs. The formula for the Getis-Ord Gi* statistic is as follows [49, 50]:

$$G_{i}^{*}=\frac{\sum_{j=1}^{n}w_{ij}x_{j}-\bar{X}\sum_{j=1}^{n}w_{ij}}{S\sqrt{\frac{\left[n\sum_{j=1}^{n}w_{ij}^{2}-{\left(\sum_{j=1}^{n}w_{ij}\right)}^{2}\right]}{n-1}}}$$ (9)

Here, x_j_ represents the observed value, w_ij_ denotes the spatial weight, $\bar{X}$ is the mean of the observed values, and S stands for the standard deviation. This method reveals the patterns of aggregation or dispersion of ESs across different spatial and temporal scales, providing a scientific basis for future land use and ecological conservation strategies.

### 2.7. Calculation of SDG based on various types of ESs

**Fig 2:**
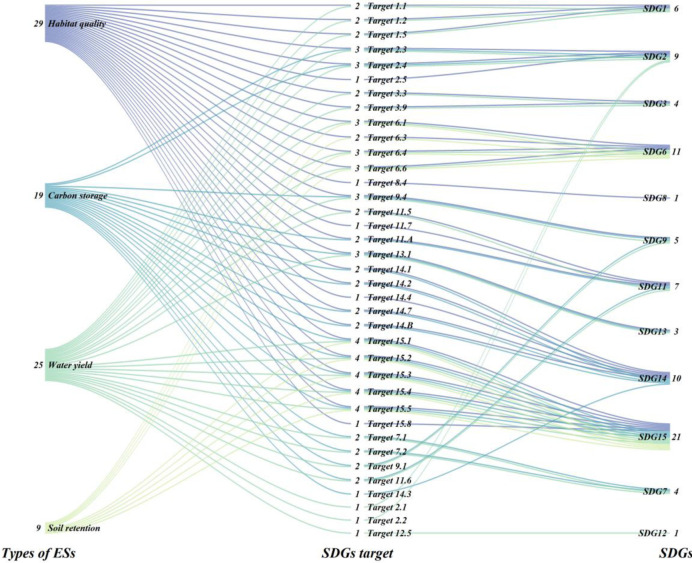
Correspondence of support for SDGs across ESs categories.

Due to the differing units of measurement for the indicators of various ESs, it is not feasible to perform additive calculations directly in the experiment. To eliminate the errors caused by these discrepancies, normalization was applied. In 2018, Wood et al. [51] proposed the connection between ESs and SDGs, highlighting how different ecosystem indicators support various SDG [52], as shown in Fig 2. Habitat quality supports 10 SDGs with a total of 29 targets, impacting areas such as poverty, food security, and ecological conservation. Carbon storage supports 7 SDGs with 19 targets, closely related to energy sustainability and climate action. Water yield supports 10 SDGs with 25 targets, addressing food and water security as well as sustainable urban development. Soil retention supports 2 SDGs with 9 targets, primarily linked to water resource management and ecological conservation. Overall, these ESs demonstrate the importance of ecosystem health for sustainable development, being indispensable in both environmental protection and socio-economic development. In this experiment, the weights of ESs indicators and SDGs indicators were both set to 1, implying equal importance in the assessment calculations. The calculation formula is as follows [53–56]:

$$SDG_{j}=\frac{\sum_{i=1}^{n}\left({\tilde{ES}}_{i}\times G_{ij}\right)}{\sum_{i=1}^{n}G_{ij}}$$ (10)

$$SDGs=\frac{\sum_{j=1}^{m}\left(SDG_{j}\right)}{m}$$ (11)

In the formula, SDGj represents the score for the jth goal, and Gij is the amount of support provided by the ith ES for the jth SDG.

## 3. Results

### 3.1. Land-use simulations based on climate change

**Fig 3:**
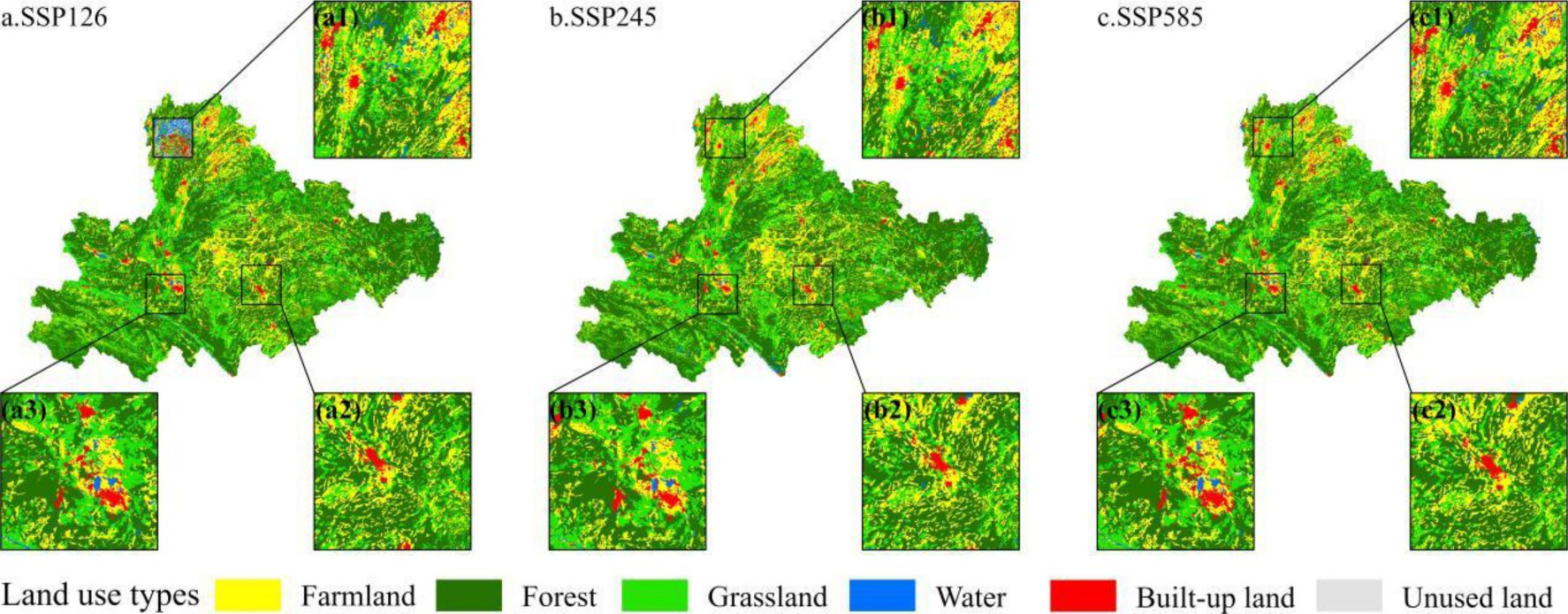
Spatial patterns of LUC in 2035 different scenarios.

The LUC trends in the karst region of southeastern Yunnan by 2035 remain consistent across different scenarios. In all three scenarios, water and build-up land show varying degrees of increase, whereas farmland, forest, grassland, and unused land exhibit different degrees of reduction. However, there are significant differences in the intensity of these changes and spatial patterns (Fig 3, Table 3). Under the SSP126 scenario, farmland protection is optimal, with a decrease of 218.29 km^2^ compared to 2020, which is lower than the 232.08 km^2^ and 240.36 km^2^ decreases seen in the SSP245 and SSP585 scenarios, respectively. Forest area decreases the least under the SSP245 scenario, with a reduction of 189.36 km^2^ compared to 2020, which is less than the reductions of 232.79 km^2^ and 265.62 km^2^ seen in the SSP126 and SSP585 scenarios, respectively. Grassland experiences significant loss, with decreases of 287.11 km^2^, 310.53 km^2^, and 285.85 km^2^ under the SSP126, SSP245, and SSP585 scenarios, respectively. Unused land decreases the least under the SSP245 scenario, with a reduction of 20.56 km^2^ compared to 2020, which is lower than the 57.40 km^2^ and 35.91 km^2^ reductions under the SSP126 and SSP585 scenarios. Water area increases the most under the SSP126 scenario, with an increase of 537.63 km^2^, significantly higher than the 249.43 km^2^ and 302.69 km^2^ increases under the SSP245 and SSP585 scenarios. Build-up land increases the most under the SSP585 scenario, with an increase of 525.05 km^2^, slightly higher than the 503.10 km^2^ under the SSP245 scenario, and significantly higher than the 257.96 km^2^ under the SSP126 scenario.

**Table 3:** LUC in southeast Yunnan under different scenarios in 2035.

Land use types	2020 year	SSP126		SSP245		SSP585	
		Areas	Change	Areas	Change	Areas	Change
**Farmland**	13991.32	13773.04	-218.28	13759.24	-232.08	13750.96	-240.36
**Forest**	38543.17	38310.38	-232.79	38353.82	-189.35	38277.55	-265.62
**Grassland**	17895.12	17608.01	-287.11	17584.59	-310.53	17609.27	-285.85
**Water**	516.38	1054.01	537.63	765.81	249.43	819.07	302.69
**Built-up land**	904.95	1162.91	257.96	1408.05	503.1	1430	525.05
**Unused land**	76.87	19.47	-57.4	56.31	-20.56	40.96	-35.91

### 3.2. Spatio-temporal evolution of ESs based on climate change

**Fig 4:**
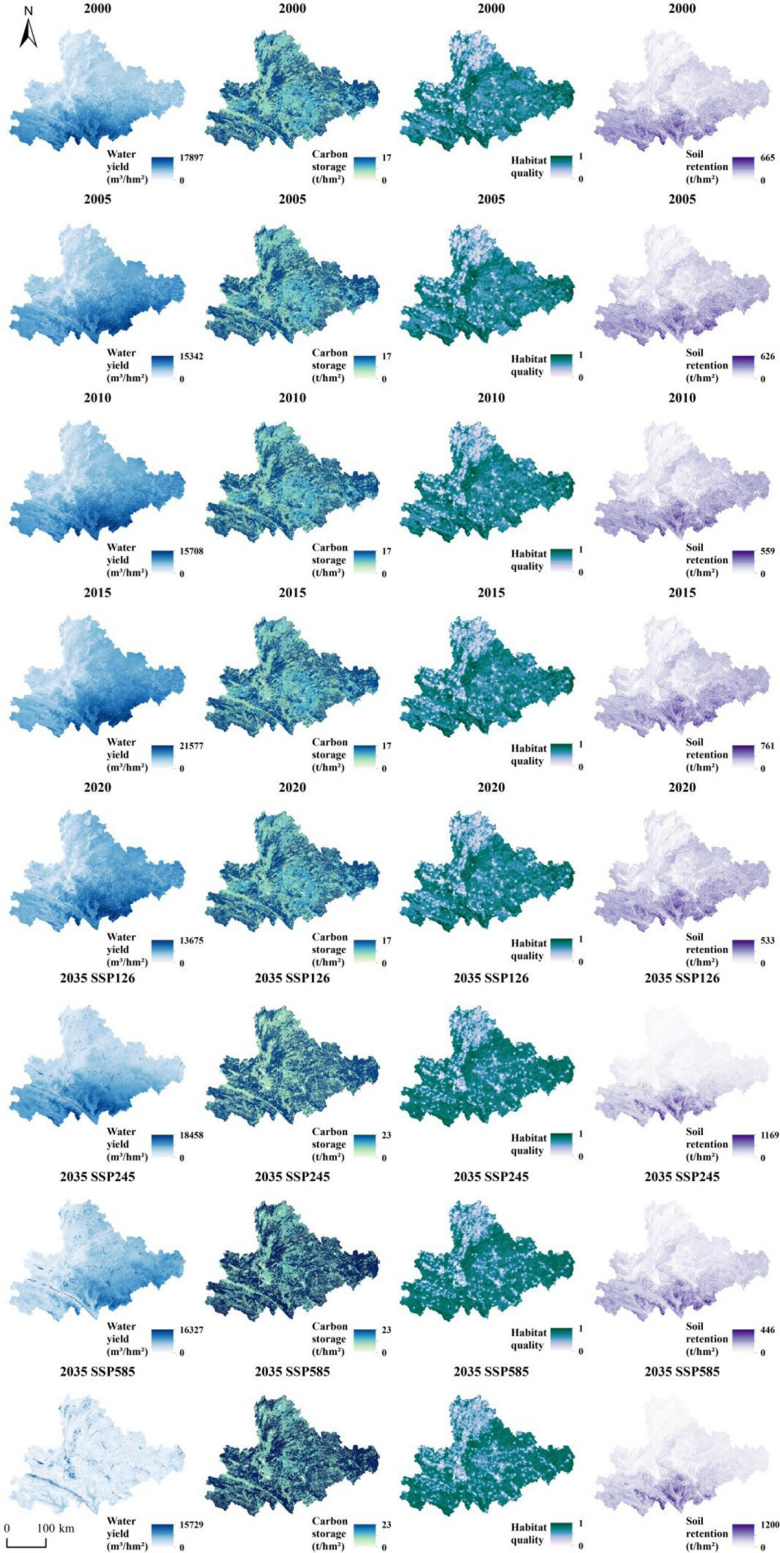
Spatial distribution of ESs.

Between 2000 and 2020, the study area’s water yield showed a pattern of higher levels in the southeast and lower levels in the northwest. By 2035, its spatial distribution remains akin to that of 2020 across different scenarios (Fig 4). When compared to 2020, the total water yield sees an increase in all three scenarios, with the SSP126 scenario experiencing the largest rise. The spatial distribution of carbon storage was dispersed from 2000 to 2020 and continues similarly in 2035 under various scenarios. Nonetheless, total carbon storage decreases across all scenarios relative to 2020, with the SSP126 scenario exhibiting the least reduction. From 2000 to 2020, habitat quality displayed a pattern of better conditions in the south and poorer conditions in the north. In comparison to 2020, habitat quality sees a decline in all three scenarios, although the SSP126 scenario shows the smallest decrease. Soil retention between 2000 and 2020 also demonstrated a south-high, north-low trend, with high-value areas being less extensive and low-value areas more prevalent. Compared to 2020, soil retention increases across all scenarios, with the SSP126 scenario showing the most significant improvement.

### 3.3. Spatial correlation of ESs under climate change

**Fig 5:**
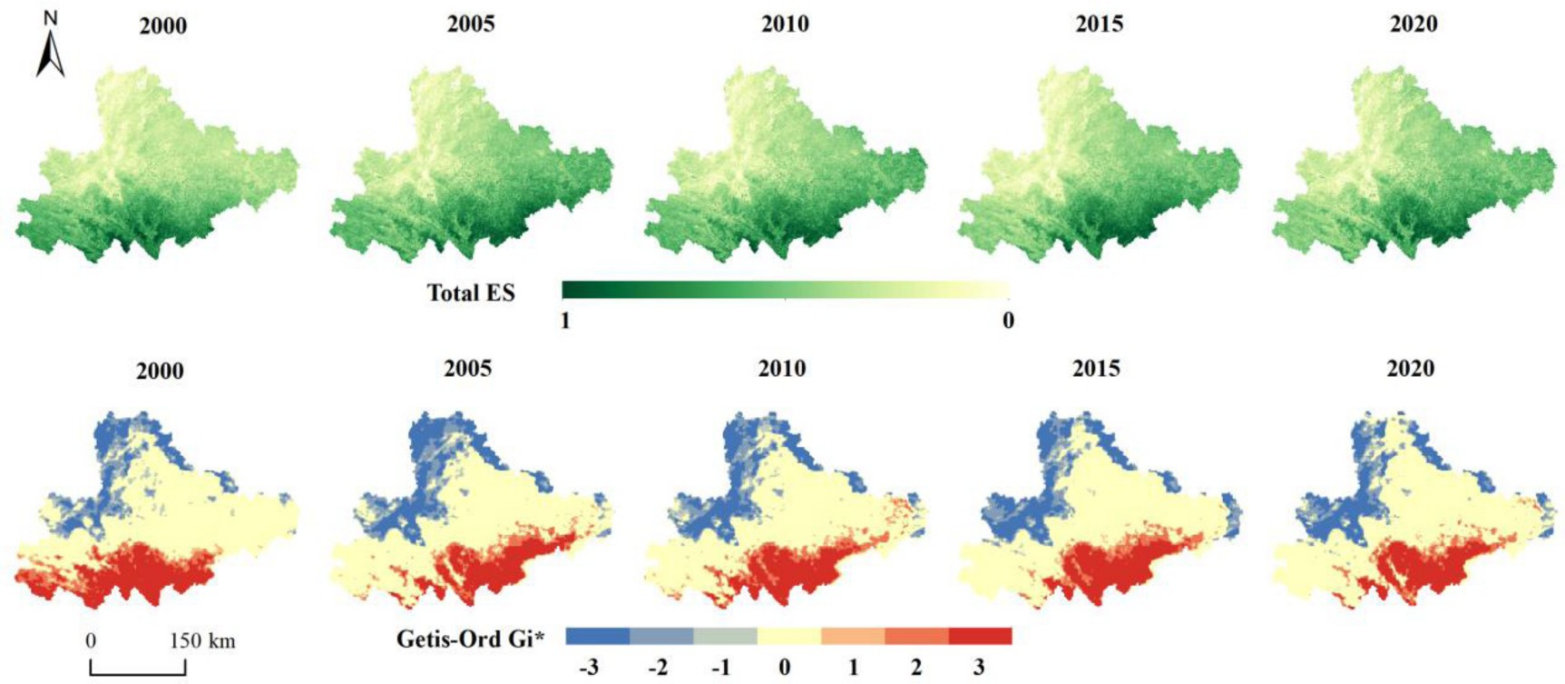
Distribution of ESs and hot and cold spots during the historical period. Notes: -3: Cold spot—99% Confidence; -2: Cold spot—95% Confidence; -1:Cold spot—90% Confidence; 0: No significant; 1: Hot spot—99% Confidence; 2: Hot spot—95% Confidence;3:Hot spot—90% Confidence, the same as below.

Using the entropy weight method, the weights for the four indicators were determined as follows: carbon storage at 0.24, habitat quality at 0.27, soil retention at 0.24, and water yield at 0.25. By aggregating these normalized indicators, we derived the total ESs. From 2000 to 2020, the spatial distribution of ecosystem services showed a pattern of "low in the northeast and high in the southwest" (Fig 5). The Moran’s I index for ESs is greater than 0, indicating a certain degree of spatial clustering. The Getis-Ord Gi* analysis revealed significant spatial differences in hot and cold spots from 2000 to 2020, with cold spots gradually expanding towards the northwest. Hot spots were primarily located in the southern and eastern remote areas of Yunnan, and by 2020, the hot spots in the eastern region also increased.

**Fig 6:**
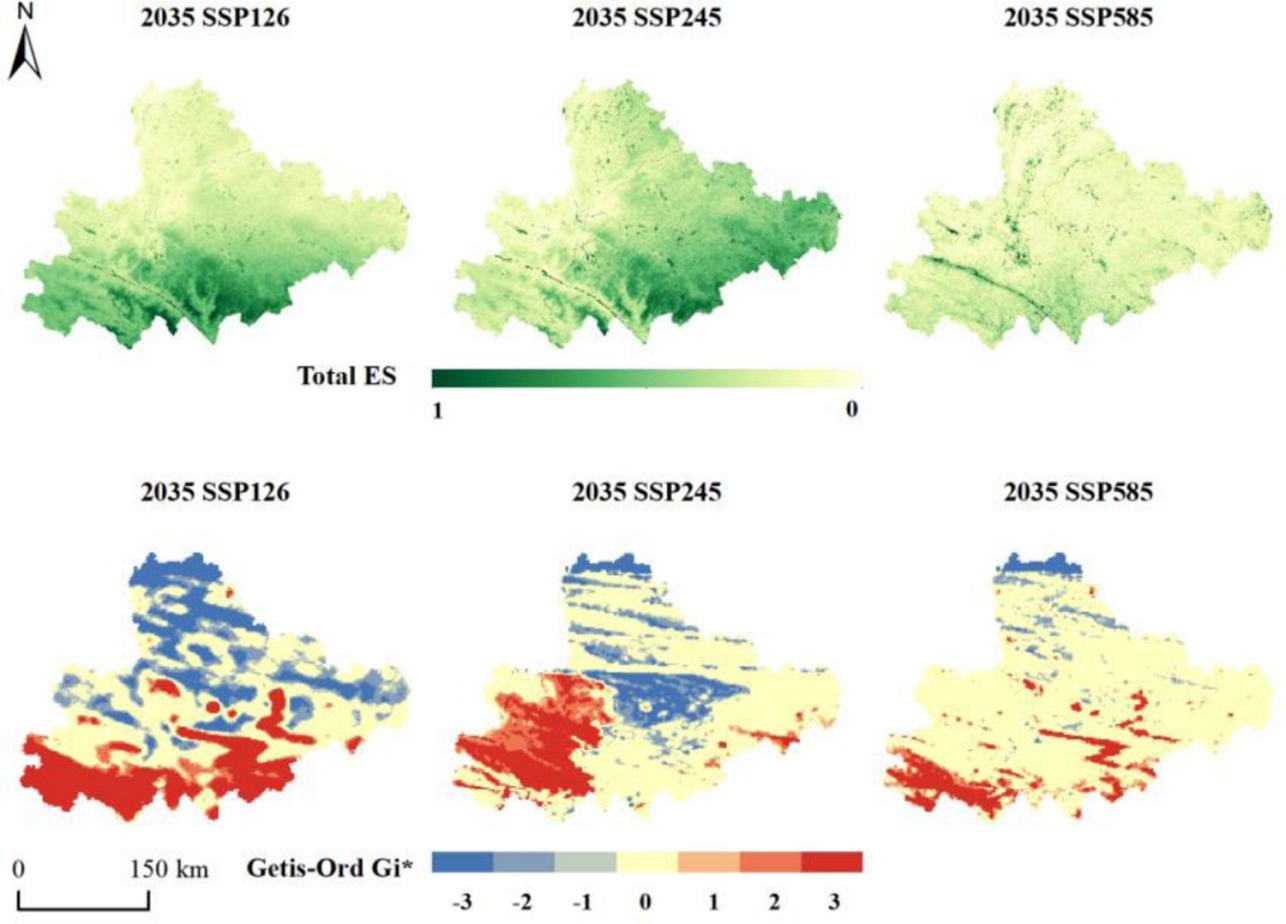
Distribution of total ESs and hotspots under future climate change.

By 2035, total ESs (Fig 6) have improved compared to 2020 across the three SSP scenarios. The highest values are located in the west and progressively decrease moving eastward. All three SSP scenarios have Moran’s I indices greater than 0, demonstrating notable spatial clustering. In the SSP126 and SSP245 scenarios, hot spots have significantly increased from 2020, forming prominent clusters. Cold spots are mainly found in the central Yunnan urban areas. For the SSP585 scenario, hot spots are more scattered, showing a fragmented spatial pattern without large continuous areas. This scenario features marked urban expansion and increased farmland, which heavily impact the ecological environment and result in an aggregation of cold spots.

### 3.4. Analysis of the spatio-temporal evolution of SDGs based on ESs

**Fig 7:**
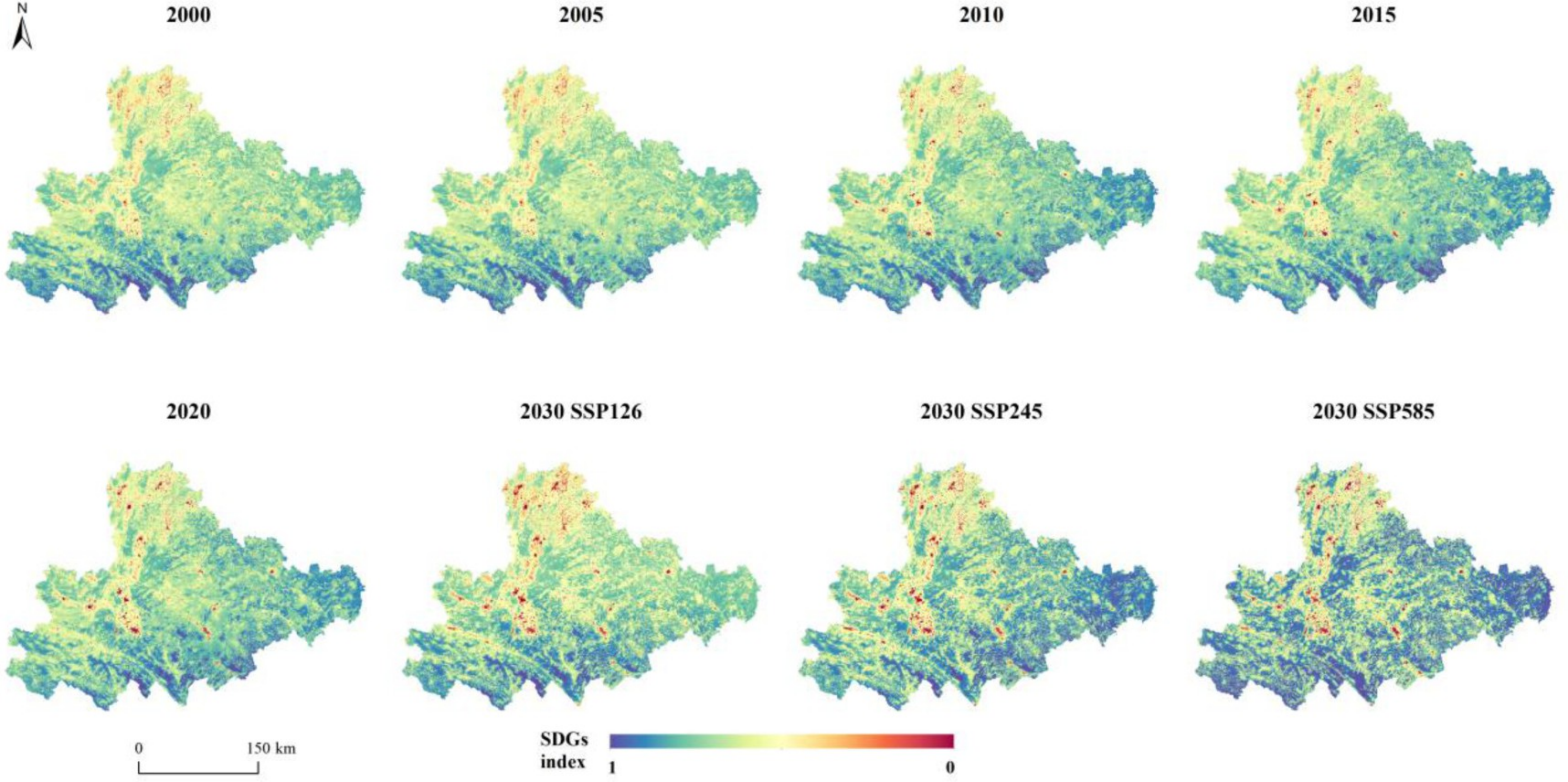
Spatio-temporal changes in SDG under multi-scenario spatio-temporal scenarios.

Based on four categories of ESs and referencing Fig 7, which illustrates the relationship between ESs and SDGs, these ESs collectively support 12 SDGs. By spatially quantifying these 12 goals, an overall SDG score was obtained. Spatially, the SDG index in the study area is generally lower in the north and higher in the south, particularly in the southeast. Between 2000 and 2020, low SDG value regions in the central-western part of the study area gradually concentrated, while high-value regions in the east increased annually. Compared to 2020, under the SSP126 scenario in 2030, the SDG index in the southern part of the study area increases, while it decreases in the eastern part. Under the SSP245 scenario in 2030, the SDG index significantly rises from central to eastern regions. By 2030, under the SSP585 scenario, the overall SDG index for the study area shows a marked increase.

## 4. Discussion

### 4.1. Analysis of changes in LUC and various types of ES under climate change

Against the backdrop of climate change, the land use types in the karst region of southeastern Yunnan have undergone significant changes, alongside varying trends in ESs. According to simulation results, the areas of farmland, forest, and grassland have generally decreased, while water and construction land have increased. This change in land use is not only a result of climate and economic development but also reflects a response to land use policies and management measures. This suggests that we need to consider the long-term impacts of climate change on land sustainability and ecological capacity in a more systematic way. Policymakers should prioritize data-driven approaches to dynamic land planning in order to balance development with environmental protection. Several key factors drive these changes. Climate change-induced temperature rise and precipitation pattern shifts directly influence regional land use patterns. Particularly under the SSP585 scenario, the significant increase in built-up land is driven by accelerated urbanization due to population and economic growth, as well as intensified human activities. This urban expansion often necessitates the conversion of more farmland and natural ecosystems into built-up land, further reducing the areas of farmland and grasslands. This trend is clearly evident in the research findings, highlighting the challenges that exist between economic development and land use sustainability. ES capacities also manifest diverse changes under different scenarios. Regarding water yield, while the total water yield in 2035 increases compared to 2020. This indicates that although climate change may enhance precipitation and water resource availability, the ecological functions of soil and vegetation might be compromised, leading to reduced carbon sequestration and declining habitat quality. It indicates that only through integrated, multidimensional management strategies can we effectively address the changes in these ES functions. For example, by enhancing forest management and wetland conservation measures, we can improve regional carbon storage capacity while maintaining water and soil functions.

The fluctuations in land use and ESs also reflect the effectiveness of policies and management measures. In the current climate and economic context, formulating and implementing effective land use policies to achieve both ecological and economic benefits is a critical future research and resolution topic. Effective land management measures can mitigate the adverse effects of climate change to some extent and promote the achievement of SDGs. Future land use policies should fully consider the interactions between climate change, ecological conservation, and economic development in order to achieve more sustainable regional development.

### 4.2. Characteristics of spatial clustering of ES and changes in SDG under climate change

In the southeastern Yunnan karst region, ESs exhibit notable spatial clustering characteristics under the influence of climate change. Particularly in the SSP126 and SSP245 scenarios, hotspots are primarily concentrated in the vegetation-rich Yunling Mountains and Xishuangbanna. This phenomenon of spatial agglomeration not only reflects the influence of natural conditions but also highlights the effectiveness and specificity of regional policy measures. Such spatial disparities indicate that differentiated policy strategies should be adopted for ecological conservation. In the context of limited resources, prioritizing the protection of areas with high ES supply and high biodiversity would be a more effective resource allocation strategy. The local ecological environment and climate conditions directly impact the spatial distribution of ESs. With rising temperatures and increased precipitation due to climate change, ecological restoration has been observed in the southern and southeastern regions, leading to significant improvements in habitat quality and water availability. Compared to the northern areas, the southern regions exhibit greater clustering of ESs due to higher precipitation and dense vegetation cover. Additionally, abundant rainfall conditions enhance biodiversity, which in turn strengthens ecosystem stability and service functions. The impact of human activities varies significantly across different regions. In northern areas like Kunming and Chuxiong, rapid urbanization and the expansion of build-up land and farmland have reduced the area of ecological land, diminishing the capacity to supply ESs. This contrasts sharply with the southern regions, where relatively lower human interference has preserved a good ecological environment, promoting the effective provision of ESs.

By 2035, under the SSP585 scenario, cold spots are concentrated in cities like Kunming and Honghe, indicating that the intensity of human activities significantly affects local ecological quality. Changes in the SDG index also reflect these spatial clustering characteristics of ESs. From 2000 to 2020, the SDG index gradually decreased in the north while remaining relatively high and increasing annually in the south. This indicates that the southern regions have made some progress in ecological protection and sustainable development. By 2035, under the SSP126 scenario, the SDG index increases in the southern areas, closely associated with the enhancement of ESs, suggesting that a better ecological environment contributes to achieving SDGs. The spatial clustering characteristics caused by climate change not only affect the distribution of ESs but also directly relate to the region’s sustainable development level. With changing climate conditions, the southeastern Yunnan karst region needs to focus more on protecting and restoring the local ecological environment to achieve better ESs and improve the SDG index, thereby promoting SDGs.

### 4.3. Recommendations and perspectives

Future research should aim to integrate a broader range of geographic information, remote sensing data, and socioeconomic data to develop more comprehensive models. Additionally, incorporating dynamic monitoring technologies can provide real-time insights into the dynamic characteristics of LUC and ESs. Only through a multi-level and interdisciplinary approach can we gain a deeper understanding of the complex impacts of climate change on regional land use and ESs. Future studies should place greater emphasis on cross-disciplinary data integration and model innovation to support more resilient decision-making. It is also essential to consider different climate change scenarios and socioeconomic development pathways to explore the impacts of various policies and management measures on ESs and sustainable development. Attention should be given to the relationship between ESs and human well-being, with quantitative assessments of the economic, social, and environmental benefits of these services enhancing public awareness and support for ecological protection. Based on research findings, local governments should craft more targeted land use management policies [57], particularly in the conservation of arable land, forests, and grasslands. Enhanced management of water resources is also crucial to address future challenges posed by climate change.

Establishing cross-sector collaboration mechanisms can foster cooperation among various fields, such as environment, agriculture, forestry, and urban planning [58]. This will help create synergies between different policies and ensure the effective implementation of land use management and ecological protection measures. Promoting an ecological compensation mechanism can encourage local governments and communities to adopt protective measures [59]. Economic incentives can guide farmers and businesses to voluntarily participate in ecological conservation activities, such as implementing eco-friendly farming practices, forest conservation, and water resource management projects, ultimately improving the capacity for ES provision [60]. Another important dimension is community participation and localized governance, which emphasizes grassroots awareness and practices for ecological conservation in order to achieve policy sustainability and broad public support. Specific guidelines and standards should be developed to promote sustainable land use practices, such as crop rotation, intercropping, and agroforestry, to reduce land degradation, enhance soil quality, and increase carbon sequestration capacity [61]. Strengthening environmental education for the public can raise awareness of the importance of ESs. Organizing community activities can encourage public participation in ecological protection and restoration projects, thus fostering a sense of environmental responsibility among local residents [62].

A multidimensional assessment framework that considers ecological, economic, and social factors will provide a more holistic perspective for land use policy formulation. In light of the uncertainties associated with climate change and socioeconomic development, future policies should be designed with greater flexibility and adaptability to respond promptly to emerging challenges and changes [63]. Learning from successful international sustainable development experiences can encourage cooperation and exchange, bringing in advanced management models and technologies to promote regional ecological protection and sustainable development [64].

## 5. Conclusion

This study explores the spatiotemporal simulation of sustainable development based on ESs in the southeastern Yunnan karst region under the backdrop of climate change. It reveals how LUCs under different scenarios impact ESs. The findings indicate that by 2035, there is a trend of increasing water bodies and construction land, whereas arable land, forests, grasslands, and unused land decrease to varying extents. Under the SSP126 scenario, the protection of arable land and forests is more effective, and the significant increase in water bodies reflects the profound impact of climate change on land use patterns. By 2035, water yield and soil retention increase across various scenarios, with the most substantial growth seen under the SSP126 scenario. However, carbon storage and habitat quality show a declining trend, especially under the SSP585 scenario, highlighting the combined pressures of human activities and climate change on the ecological environment. The overall SDG index is higher in the southern region and lower in the northern region, indicating an issue of uneven regional development. The changes in the SDG index across different scenarios for 2030 reflect the potential trends in future sustainable development.
